# Immunohistochemical Expression of *Programmed Death Ligand-1 (PDL-1)* in Colorectal carcinoma and Its Correlation with Stromal Tumor Infiltrating Lymphocytes

**DOI:** 10.31557/APJCP.2020.21.1.225

**Published:** 2020

**Authors:** Mona ELfishawy, Solafa Amin Abd-ELaziz, Azza Hegazy, Dina F EL-yasergy

**Affiliations:** 1 *Specialist of Pathology, Egyptian Ministry of Health, *; 2 *Professor of Pathology, *; 4 *Lecturer of Pathology, Faculty of Medicine, Cairo University, *; 3 *Profesor and Head of Pathology Department, National Hepatology and Topical Medicine Research Istitute, Egypt. *

**Keywords:** Colorectal carcinoma, PDL-1, CRC-PDL-1

## Abstract

**Objectives:**

Detection of Immunohistochemical (*IHC*) expression of *PDL-1* by tumor cells and stromal tumor infiltrating lymphocytes (TILs) in colorectal carcinoma, to investigate the possibility of using it as a targeted therapy, as well as, correlation of this expression with the clinico-pathologic parameters of the tumors.

**Materials and Methods:**

Colorectal tissue sections were collected from 60 colectomy specimens were taken from Kasr El Ainy Hospital, Faculty of Medicine, Cairo University. Exclusion criteria included cases with missing data and cases who received chemotherapy or radiotherapy. IHC expression of *PDL-1* was investigated in tumor cells (T) and stromal TILs separately. *PDL-1 *positivity was defined as *PDL-1* expression on ≥ 5% of membranous positive cell staining of any intensity.

**Results:**

P*DL-1 (T)* expression was detected in 25% of cases and showed statistically significant correlation with higher tumor grade and right sided colon tumors (P value < 0.05). *PD-L1* stromal *TILs* expression was detected in 38.3 % of cases. Insignificant statistical relation between Stromal *TILs* *PDL-1* expression and the tumor extent (T) was detected (P value = 0.07), however, the expression of* PDL-1* in lymphocytes was inversely proportional to the tumor extent (invasion). There were linear relation between *PDL-1* expression stromal (*TILs*) (33.3%) and *PDL-1* expression in tumor cells (28.2%) and positive lympho-vascular invasion but it was statistically insignificant (P value = 0.4 and 0.2 respectively). Despite there were no statistical relation between either *PDL-1 (T)* and *PDL-stromal TILS* and Perineural invasion (P value =1 and 0.5) but inverse relation was noticed with more *PDL-1* expression in tumor cells (24.5%) and *TILS *(40.8%) with negative Perineural invasion.

**Conclusion:**

Our results supported* PDL-1* expression in CRC by both TC and TILs, with higher expression in subset of tumors that are high grade highlighting them as candidates for anti- *PD-1/PDL-1* therapy.

## Introduction

CRC is the third most common cancer worldwide after lung and breast cancers with two-thirds of all CRCs occurring in the more developed regions of the world (Gado et al., 2013). In Egypt, CRC accounts for 6.47% of all cancers according to the National Cancer Institute, Cairo University (Mokhtar et al., 2016).

The development of CRC is a complex and heterogeneous process arising from an interaction between multiple etiological factors, including genetic factors and environmental factors, such as diet and lifestyle (Ioannou et al., 2015). 

Although the classic T.N.M. (tumor-nodes-metastasis) classification is somewhat useful for the staging of CRC patients and their selection for specific treatment, it is not a completely adequate method because patients with disease at the same stage may have different clinical outcomes, thus rendering the conventional staging system incapable of precisely predicting prognoses. Therefore, there is a great need to identify the molecular markers of more aggressive CRC in order to select patients for adjuvant systemic or targeted therapies (Kwon et al., 2010).

The immune system recognizes cancer cells as soon as they emerge. The interaction between T cells and antigen-presenting cells (APC) is complex and involves the T cell receptor and multiple co-regulatory receptors, which exert both activating and inhibitory stimuli to the T cell (Muenst et al., 2014). Despite the large number of tumor antigens induced by genetic and epigenetic changes found in all cancers, tumors resist immune attack by inducing tolerance among tumor-specific T cells and by expressing ligands that engage inhibitory receptors and dampen T-cell functions (Brahmer et al., 2012).

The programmed death (PD) 1 pathway is a major immune response checkpoint and target for cancer immunotherapy (Cimino-Mathews et al., 2015). PD-1 is a member of the B7-CD28 family of co-regulatory molecules expressed by activated lymphocytes (Cimino-Mathews et al., 2016). The interaction between PD-1 and its ligands inhibits T-cells, blocking immune responses (Baptista et al., 2016). 

Multiple solid tumor types including melanoma, renal cell carcinoma, non-small cell lung carcinoma, thymoma, ovarian, and colorectal cancer co-opt this immune shield by expressing *PDL-1* to generate an immunosuppressive tumor microenvironment and avoid T cell cytolysis. Upregulated* PDL-1* binds PD-1 on T cells, contributing to the development of T-cell exhaustion. Tumor cells have co-opted this *PD-1/PDL-1* regulatory mechanism, normally designed to prevent autoimmune attacks, and instead overexpress *PDL-1* to avoid immunologic surveillance and to facilitate cancer growth (Patel and Kurzrock, 2015). These properties make *PDL-1* a potentially promising target for cancer immunotherapy (Brahmer et al., 2012). Where antibodies targeting either PD-1 or *PDL-1* have shown durable, objective responses in patients with highly immunogenic tumors such as melanoma, non-small-cell lung cancer and renal-cell carcinoma (Topalian et al., 2012).

## Materials and Methods

A total of 60 colectomy specimens of patients with colorectal adenocarcinoma were taken from Kasr El Ainy Hospital, Faculty of Medicine, Cairo University, in the period from December 2016 through March 2017. The tumor sections were dissected, then formalin fixed and paraffin embedded. The clinical data of these cases including age and sex were taken from their pathology requisition sheets enclosed with the specimens. The specimens were anonymous for confidentiality and replaced by numbers.

Inclusion criteria included any case of colorectal cancer had colectomy specimen.

Exclusion criteria included cases with missing data and cases who received chemotherapy or radiotherapy.

Each paraffin block was re-cut by rotatory microtome at 4-5 microns thickness then mounted on glass slides and stained by hematoxylin and eosin (H&E) for routine histopathological examination and on charged slides for immunostaining.

The routine pathological examination included morphologic classification of the colorectal carcinoma according to the recommendations of the World Health Organization (Hamilton et al., 2010), staging was performed using modified Dukes’ classification of the disease (Bresalier, 2010). The TNM staging was applied according to the American Joint Committee of Cancer (AJCC) and the International Union for Cancer Control (UICC) (Jessup J.M. et al., 2017). 

*Immunohistochemistry*

The slides were put in Dako autostainer which performed the rest of the steps as follows; Incubation in 3% H_2_O_2_ for 5 minutes to inhibit the endogenous peroxidase activity. Washing the slides with phosphate buffered saline (PBS) at Ph 7.2-7.4. The slides were incubated with BIOCARE medical monoclonal rabbit anti-human* PDL-1* 1:100 in DAko antibody diluents S3022 for 30 minutes at room temperature. Washing the slides with phosphate buffered saline (PBS) at ph 7.2-7.4. Applying the Envision Dako link kit optimized for Dako cytomation automated system for 30 minutes. Washing the slides with phosphate buffered saline (PBS) at Ph 7.2-7.4. Applying DAB (3,3’-di-amino-benzidinetetrahydrochloride) as chromogen for 5 minutes. The slides were rinsed well in distilled water for 5 minutes.The slides in the autostainer were removed and Meyer’s Hematoxylin counter stain was performed. Slides were dehydrated in ascending grades of alcohol and were cleared in xylene for 3 changes and cover slips were applied.

A section of tonsil was used as positive control according to the manufacturer recommendations.

*Evaluation*

*PDL-1* immunohistochemical staining was scored in both the tumor cells (T) and the stromal TILs.

*PDL-1 *immunoreactivity was evaluated separately for tumor cells and stromal TILs. *PDL-1* positivity was defined as *PDL-1* expression on ≥ 5% of membranous positive cell staining of any intensity (Valentini et al., 2018). 

Cytoplasmic staining was not considered in this study, only membranous staining was considered (Xing et al., 2017) as *PDL-1* localization to the cell membrane is likely required for interaction with PD-1 (Rebelatto et al., 2016). In addition, the FDA approved antibodies for PDL-1 testing in the lung cancer (Hirsch et al., 2017) and urothelial cancer (Roche receives FDA approval,” 2017), they interpret it only in the cell membrane.

Another pattern of staining was noticed while examining the slides which was cytoplasmic granular and it was considered negative (Phillips et al., 2015). 

Statistical Analysis: The previously mentioned clinical, histopathological and immunohistochemical data was then transferred to the Statistical Package of Social Science (SPSS) Software program, version 25 to be statistically analyzed. Comparison between groups was then performed using Chi square test. A P value of ≤ 0.05 was considered statistically significant and of ≤ 0.01 were considered highly significant. Graphs were used to illustrate some information.

All slides were screened using a Leica DM500 microscope. Microscopic photos were captured using a digital camera attached to an Olympus microscope model BX 51.

## Results

This study included 60 cases of colorectal adenocarcinoma with a mean age 57.5 years. 58.3 % of the cases were males and 41.7 % were females. Colonic tumors represented 68.3% of the cases with more prevalence in left sided one (38.3%) and rectal tumors represented 31.7% of cases. All cases were adenocarcinoma (100%). 6.7% of the cases were G1, 75% were G2 and 18.3% were G3. 70% of the cases were T3. 21% of the cases showed no nodal metastasis. 65% of the cases showed positive lympho-vascular invasion and 18.3% of cases showed Perineural invasion. 30% of the cases were stage group IIIB. The pathological data of the cases are summarized in Table 1.

**Figure 1:**
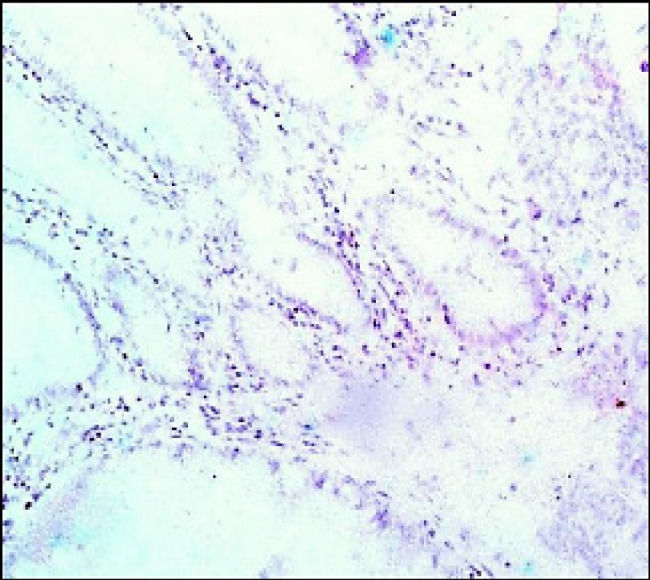
Colorectal Adenocarcinoma GI with Absent immunohistochemical Expression of *PDL-1* (Original Magnification, x100).

**Figure 2:**
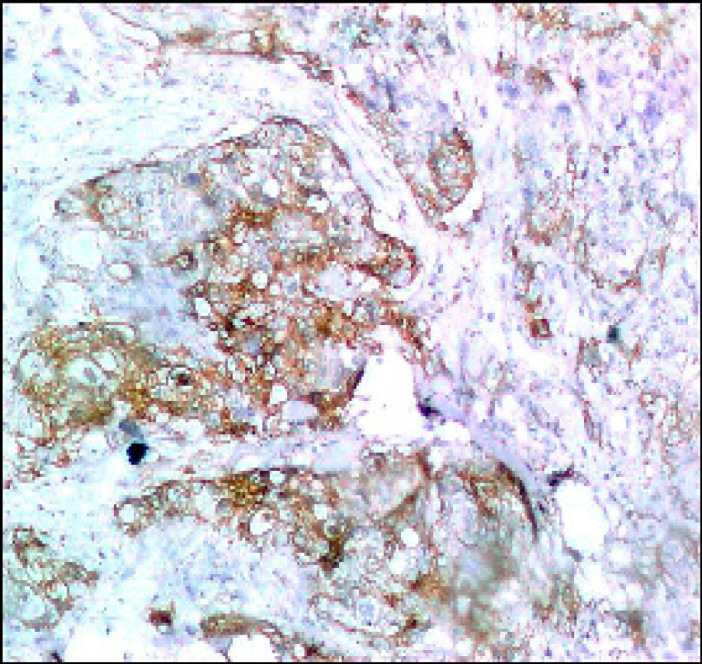
Colorectal Adenocarcinoma GII Showing Membranous PDL-1 Immunostaining of Tumor Cells (Original Magnification, x200).

**Figure 3:**
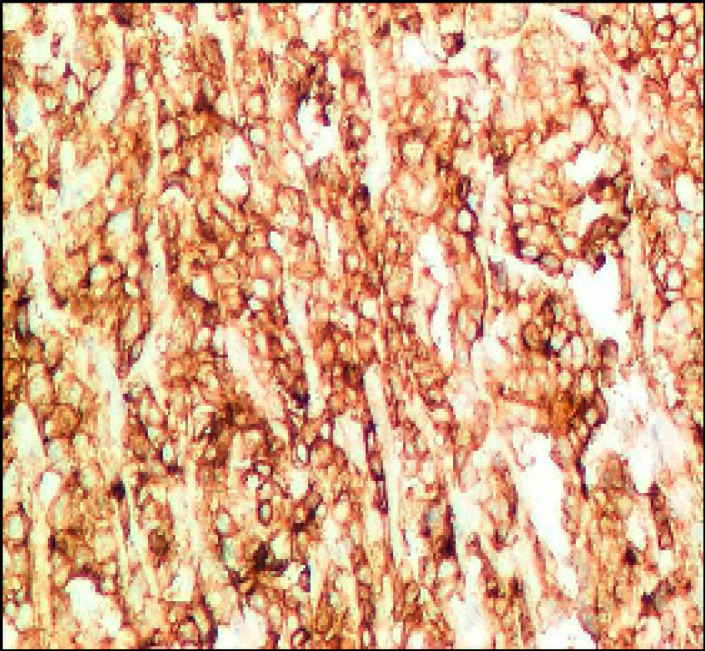
Colorectal Adenocarcinoma GIII Showing Sheets of Malignant Cells with Membranous Immunostaining of *PDL-1* (Original Magnification, x200).

As regards *PDL-1 (T)* expression, it was positive in 15 cases [28%] and negative in 45 cases [72%]. All G1 cases were negative Figure 1, 8.8% of G2 were positive Figure 2 and 100% of G3 cases were positive Figure 3.

**Figure 4:**
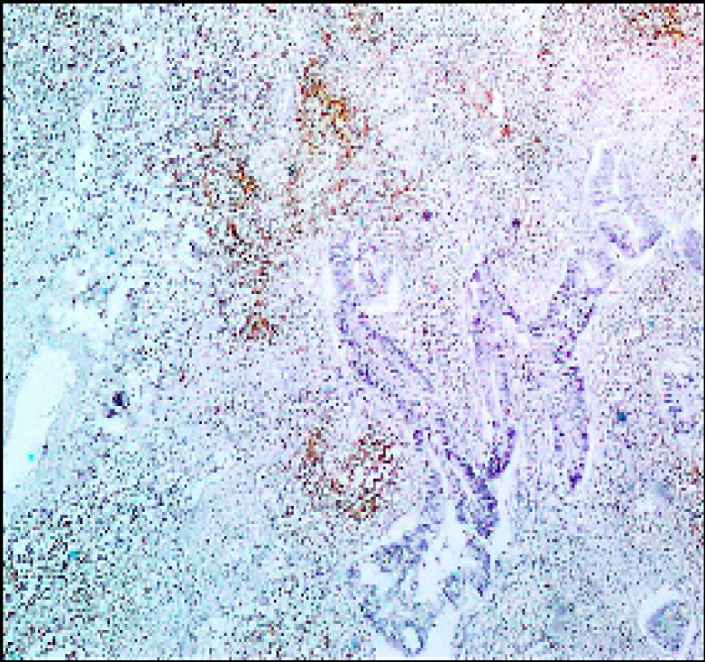
Colorectal Adenocarcinoma GII Showing Membranous PDL-1 Immunostaining of Lymphocytes (Original Magnification, x100).

PDL-1 Stromal TILs expression was positive in 23 cases [38.3%] and negative in 37 cases [61.7%] Figure 4.

**Figure 5:**
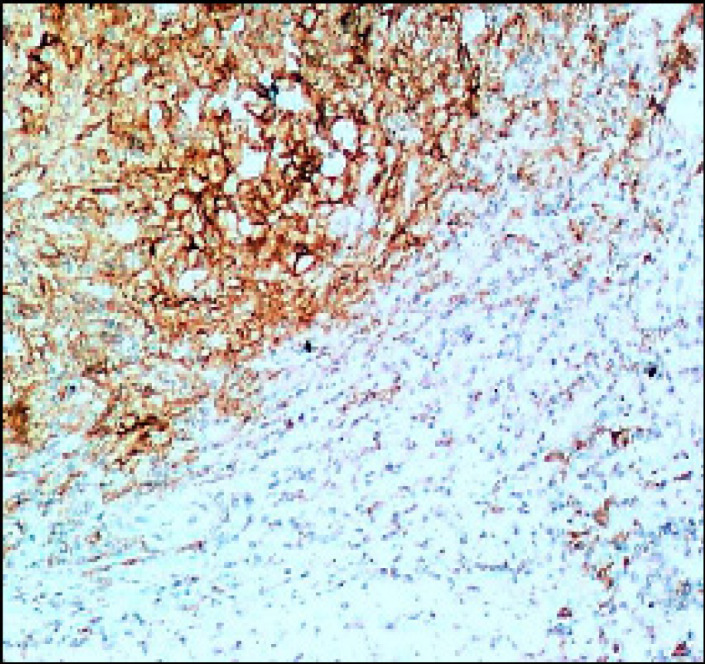
Colorectal Adenocarcinoma GII Showing Membranous PDL-1 Immunostaining of both Tumor Cells and Stromal TILs (Original Magnification, x100)

Only 8 [13.3%] cases showed overlapped *PDL-l (T) *and *Stromal TILs* expression Figure 5.

No significant relation was found between gender and *PDL-1* expression in (T) and (TILs) [P value = 0.65 and 0.44 respectively].

*PDL-1 (T)* expression showed statistically significant correlation with right sided colon tumors (P value =0.03), but on the other hand, *PDL-1 (TILs)* expression showed no significant relation with the site of the tumor (P value=0.8).

A highly statistically significant direct correlation was detected between histological grade and PDL-1 expression in tumor cells [P value =<0.01] meanwhile no correlation was detected between the histologic grade and *PDL- 1* expression in *TILS* (P value = 0.5).

The relation between the tumor extent (T) and *PDL-1* expression in tumor cells showed no significant relation (P value= 0.4). Although the relation between Stromal *TILs PDL-1* expression and the tumor extent (T) was statistically insignificant (P value = 0.07), it was noticed that the expression of *PDL-1* in lymphocytes was inversely proportional to the tumor extent (invasion). 

The relation between *PDL-1* expression stromal (TILs), PDL-1 expression in tumor cells and lympho-vascular invasion was statistically insignificant (P value = 0.4 and 0.2 respectively) but there were linear relation with positive lympho-vascular invasion. 

Despite there were no statistical relation between both *PDL-1 (T)* and *stromal TILS* with Perineural invasion (P value= 1 and 0.5) but inverse relation was noticed with more PDL-1 expression in tumor cells and *TILS* with negative Perineural invasion. All data regarding the correlation parameters of *PDL-1 (T) *and* TILS* were summarized in Table 2. 

**Table 1:** The Pathological Data of the Collected Cases

Parameter			Number (%)
Sex	Female Male		25 (41.7%) 35 (58.3%)
Site	Colon	Right Left	18 (30%) 23 (38.3%)
	Rectum		19 (31.7%)
Histological type	Adenocarcinoma		60 (100 %)
Grades of differentiation of adenocarcinoma	G1 G2 G3		4 (6.7%) 45 (75%) 11 (18.3%)
Extent of primary tumor	T1 T2 T3		1 (1.7%) 4 (6.7%) 42 (70%)
	T4	A B	6 (10%) 7 (11.6%)
Lymph node	0		21 (35%)
	1	A B	2 (3.3%) 18 (30%)
	2	A B	8 (13.3%) 11 (18.3%)
Distant metastasis	M0 M1		57 (95%) 3 (5%)
Lympho-vascular invasion	Negative Positive		21(35%) 39 (65%)
Perineural invasion	Negative Positive		49 (81.7%) 11 (18.3%)
Stage Group	I		3 (5%)
	II	A B C	14 (23.3%) 2 (3.3%) 1 (1.7%)
	III	A B C	3 (5%) 18 (30%) 16 (26.7%)
	IV A		3 (5%)
Modified Duke's	B	1 2	3 (5%) 18 (30%)
	C	1 2	2 (3.3%) 33 (55%)
	D	3	1 (1.7%)
			3 (5%)

**Table 2:** Correlation between PDL-1 in Tumor Cells, Stromal TILs and Clinico-pathological Parameters

			PDL-1 (T) +ve	PDL-1 (T) -ve	P value	PDL-1 (TILs)+ve	PDL-1 (TILs)-ve	P value
Sex	Male		8 (22.9%)	27 (77.1%)	0.65	12 (34.3%)	23 (65.7%)	0.44
	Female		7 (28%)	18 (72%)		11 (44%)	14 (56%)	
Site	Colon	Right	8 (44.4%)	10 (55.6%)	0.03*	8 (44.4%)	10(55.6%)	0.8
		Left	2 (8.7%)	21 (91.3%)		8 (34.8%)	12(63.2%)	
	Rectum		5 (26.3%)	14 (73.7%)				
Histological grade	G1		0 (0%)	4 (100%)	<0.01*	0 (0%)	4 (100%)	0.5
	G2		4 (8.8%)	41 (91.1%)		18 (40%)	27 (60%)	
	G3		0 (0%)	11 (100%)		5 (45.5%)	6 (54.5%)	
Tumor extent	T1		1 (100%)	0 (0%)	0.4	1 (100%)	0 (0%)	0.07
	T2		0 (0%)	4 (100%)		2 (50%)	-50%	
	T3		11 (26.2%)	31 (73.8%)		17 (40.5%)	25 (59.5%)	
	T4		3 (23.1%)	10 (76.9%)		3 (23.1%)	10 (76.9%)	
Lymph node status	0		6 (28.6%)	15 (71.4%)	0.6	10 (47.6%)	11 (52.4%)	0.6
	1		A	1 (50%)		0 (0%)	2 (100%)	
			B	15 (83.3%)		7 (38.9%)	11 (61.1%)	
	2		A	5 (62.5%)		3 (37.5%)	5 (62.5%)	
			B	9 (81.8%)		3 (27.3%)	8 (72.7%)	
Distant metastasis	M0		14 (24.6%)	43 (75.4%)	1	22 (38.6%)	35 (61.4%)	1
	M1		1 (33.3%)	2 (66.7%)		1 (33.3%)	2 (66.7%)	
Lymphovascular invasion	Positive		11 (28.2%)	28 (71.8%)	0.4	13 (33.3%)	26 (66.7%)	0.2
	Negative		4 (19%)	17 (81%)		10 (47.6%)	11 (52.4%)	
Perineural invasion	Positive		3 (27.3%)	8 (72.7%)	1	3 (27.3%)	8 (72.7%)	0.5
	Negative		12 (24.5%)	37 (75.5%)		20 (40.8%)	29 (59.2%)	
Stage	I		1 (33.3%)	2 (66.7%)	0.8	2 (66.7%)	1 (33.3%)	0.6
	II		A	9 (64.4%)		7 (50%)	7 (50%)	
			B	2 (100%)		0 (0%)	2 (100%)	
			C	1 (100%)		0 (0%)	1 (100%)	
	III		A	3 (100%)		2 (66.7%)	1 (33.3%)	
			B	13 (72.2%)		7 (38.9%)	11 (61.1%)	
			C	13 (81.2%)		4 (25%)	12 (75%)	
	IV A		1 (33.3%)	2 (66.7%) S		1 (33.3%)	2 (66.7%)	
Modified Duke's	B		1	2 (66.7%)	1	2 (66.7%)	1 (33.3%)	0.5
			2	13 (72.2%)		8 (44.4%)	10 (55.6%)	
	C		1	2 (100%)		1 (50%)	1 (50%)	
			2	25 (75.8%)		10 (30.3%)	23 (69.7%)	
			3	1 (100%)		1 (100%)	0 (0%)	
	D		1 (33.3%)	2 (66.7%)		1 (38.3%)	2 (61.7%)	

## Discussion

Immunotherapy targeting immune checkpoint has emerged as a promising therapeutic strategy against cancer (Inaguma et al., 2017). *PDL-1* has been reported to function in the immunoregulatory system during certain conditions, including autoimmune disease, allograft rejection, pregnancy, and cancer. Several studies suggested that PDL-1 expression in lymphocytes and in tumor cells of CRC is related to a high density of tumor infiltrating immune cells (Liu et al., 2018).

Anti-PDL-1 therapy is one of the immunotherapies to treat cancer (especially solid tumor). *PD-L1* expression may be associated with efficacy of anti *PD-1/PDL-1* therapy (Guo et al., 2017).

*PDL-1* Expression in the tumor cells was in 25% of the cases and that was compatible with the results of Valentini (2018) who exact the same result for* PDL-1* expression in tumor cells. Other studies showed nearer values as Gatalica et al., (2014) by 21% and Eljabbour et al., (2017) by 29%. Others studies showed either low figures as Rosenbaum et al., (2016) 9% and Kim et al., (2016) 12.5% or high figures as Zhong et al., (2018) 79.3% and Masugi et al., (2016) 89%. 

*PDL-1* expression in immune cells was positive in 23 cases (38.3%) and negative in 37 cases (61.7%). Kim et al., (2016) showed near result by 29.8 % for positive cases. Different results were also scored as 5% by Masugi et al., (2016) and 78% by Valentini et al., (2018) for positive cases.

*PDL-1* expression in both (T) and (*TILs*) was detected in 8 cases (26.7%) with near similar value in Valentini et al., (2018) study (25.4 %)**.** However, Wang et al., (2016) and Kim et al., (2016) proposed that they rarely overlap by 4% and 5.8% respectively.

In the current study, no statistical significant correlation between *PDL-1* rate of expression in both (T) and (*TILs*) and some of the clinicopathological parameters such the Duke’s classification and distant metastasis (p-value>0.05).

In the present study, there was no relation between *PDL-1* expression in both (T) and (*TILs*) with the gender. This is also stated by Masugi et al., (2016) and Valentini et al., (2018) in their studies. However, Lee et al., (2016), Kim et al., (2016) and Rosenbaum et al., (2016) found positive correlation between the *PDL-1* expression in tumor cells and female gender by 74%, 73% and 81% respectively.

In the current study, *PDL-1* expression in tumor cells was positively correlated with right sided tumors (44.4%) compatible with Lee et al., (2016) who stated that *PDL-1 *expression was higher in right colonic tumors (63%) than left sided one (37%), Inaguma et al., (2017) (46% in right sided tumors, 15% in transverse, 31% in left side tumors and 9% in rectum) and Valentini et al., (2018) (81.25% in right side tumors and 18.75% in left side tumors). However, Enkhbat et al., (2018) found a relation between *PDL-1 *and left sided tumors (53.8%) while Liu et al., (2018) showed that the relation was with rectal tumors (68%).

There was no relation between *PDL-1* expression in lymphocytes and tumor location in the present study, findings compatible with Masugi et al., (2016) but Valentini 2018 stated that there was a relation between *PDL-1* expression in lymphocytes and right sided tumors (59.18%) than left sided tumors (40.82%).

Histological grade was significantly correlated with *PDL-1* expression in tumor cells (P value <0.01) where all poorly differentiated tumors (G3) were positive (100%) and all the well differentiated ones (G1) were negative (100%). These findings were similar to Kim et al., (2016) documenting that 65% of positive cases were G3 while 35% were G1-2, Inaguma et al., (2017) (52% G3 and 48% G1-2), Valentini et al., (2018) (100% G3 and 0% G1-2) and Zhong et al., (2018) the positive rate in low differentiation group was higher than that of medium and high differentiation group (71.4% vs. 67.3% vs. 61.9%). Droeser et al., (2013) stated that it was correlated with low grade where G1-2 was 93.1%. However, Masugi et al., (2016) and Wang et al., (2016) stated in their studies that there no correlation between the histological grade and *PDL-1 (T)* expression. Also in Immune cells, although no statistical significance detected but all G1 tumors showed negative (100%) expression and half the G3 cases were positive (50%). These findings were similar to Valentini et al., (2018) who highlighted that despite that there was no significant statistical correlation between the *PDL-1* in (I) and the grade but the number of G3 positive cases (53.06%) were higher than G1+ G2 positive cases (46.94%). Kim et al., (2016) and Inaguma et al., (2017) stated that there was no relation between histological grade and *PDL-1* (I) expression.

In our study, no statistical significance was detected between tumor extent (T) and *PDL-1* expression in both (T) and (*TILs*) but it was noticed that the expression of PDL-1 in lymphocytes was inversely proportional to the tumor extent (invasion)100% of T1, 50% of T2, 40.5% of T3 and 23.1% of T4 were positive. Similarly, Wang et al., (2016), Rosenbaum et al., (2016), Masugi et al., (2016), Enkhbat et al., (2018) and Zhong et al., (2018) documented that there is no relation between (T) and PDL-1 (T) and (I). El Jabbour et al., (2017) documented relation between PDL-1 expression in tumor cells and late (T) stage) p value=0.036) while Droeser et al., (2013) stated that there is a relation with early (T) stage (p value 0.002).

In this study, lymph node status was also not related to *PDL-1* Expression in either (T) and (*TILs*) but Droeser et al., (2013) concluded that there was statistical relation between expression of* PDL-1* in tumor cells and absence of lymph node metastasis (p value 0.015), however, Lee et al., (2016), Rosenbaum et al., (2016), Masugi et al., (2016) and Zhong et al., (2018) supported our results (p value=0.067, 0.32, 0.14 and 0.261) respectively and also Valentini (2018) with no available p value figure. ELjabbour et al., (2017) stated that there was positive statistical relation between PDL-1 expression in tumor cells and higher lymph node status (p=0.006) but no relation with the expression in lymphocytes. 

Regarding the relation between LVI and PDL-1 expression in tumor cells in the current study, the relation was statistically insignificant but more expression was seen in cases with positive lympho-vascular invasion (73.3%). Droeser et al., (2013) and Kim et al., (2016) documented positive relation between LVI and* PDL-1 *expression in tumor cells (p value 0.017 and 0.012) respectively and that may be proved on larger sample size. Similarly we noticed slightly more expression of *PDL-1* in lymphocytes in cases with Positive LVI but Kim et al., (2016) concluded no relation between* PDL-1* expression in lymphocytes and LVI. Rosenbaum et al., (2016) and Enkhbat et al., (2018) said also there was no relation between expression of *PDL-1* and LVI.

Regarding AJCC staging system, controversial results regarded. Our study documented no relation between *PDL-1* expression in both (T) and (I) and the stage. On the contradiction, Kim et al., (2016) stated that there was positive relation between advanced stage and *PDL-1* expression in both (T) and (I). ELjabbour et al., (2017) proved that there was positive relation between advanced stage (p value 0.022) and tumor cells expression but no relation with lymphocyte expression. Although Wang et al., (2016), Lee et al., (2016), Rosenbaum et al., (2016), Masugi et al., (2016), Enkhbat et al., (2018) and Zhong et al., (2018) supported our results.

Finally, our study as well as Rosenbaum et al., (2016) and Masugi et al., (2016). documented that there was no statistical significance between the expression of *PDL-1* expression in tumor cells and lymphocytes. But ELjabbour et al., (2017) hypothesized that there was positive relation between the expression of* PDL-1* in both tumor cells and lymphocytes.

The contradictory results can be due to several factors; technical factors, such as the type of antibody, differences in immunostaining method, type of tissue blocks and it could be explained by that the immunohistochemistry was done on tissue sections, while most of the studies were carried on tissue microarrays (TMA). The limited amount of TMA tissue could underestimate focal expression of *PDL-1*.

Also assessing PDL-1 expression is complicated by many challenges: different immunohistochemical assays (i.e. different primary antibodies and assays conditions) and different *PD-L1* evaluation methods (i.e. different scoring methods and *PD-L1* positivity cut-offs) complicate the comparative evaluation between clinical studies (Scognamiglio et al., 2016).

Finally we concluded that our results are in favor with targeting the *PD-1/PD-L1* pathway as route for immunotherapy in colorectal cancer, where, *PD-L1* expression was detected in both tumor cells and lymphocytes.

*PDL-1 *showed statistically significant higher rate of expression in tumor cells of the colon than in rectum.

*PDL-1* showed higher rate of expression in poorly differentiated tumors rather than well and moderate differentiated tumors.

*PDL-1* rate of expression in tumor cells and lymphocytes showed apparent direct correlation with lympho-vascular invasion. 

*PDL-1* expression was more in both tumor cells and lymphocytes in cases with negative perineural invasion.

*PDL-1* expression in stromal infiltrating lymphocytes was inversely proportional to tumor invasiveness.

## Recommendation of The Study

One important recommendation is the correlation with the patient’s prognosis and survival.

## References

[B1] Baptista MZ, Sarian LO, Derchain SF, Pinto GA, Vassallo J (2016). Prognostic significance of PD-L1 and PD-L2 in breast cancer. Hum Pathol.

[B2] Brahmer JR, Tykodi SS, Chow LQ (2012). Safety and activity of anti–PD-L1 antibody in patients with advanced cancer. N Engl J Med.

[B4] Cimino-Mathews A, Foote JB, Emens LA (2015). Immune targeting in breast cancer. Oncology (Williston Park).

[B5] Cimino-Mathews A, Thompson E, Taube JM (2016). PD-L1 (B7-H1) expression and the immune tumor microenvironment in primary and metastatic breast carcinomas. Hum Pathol.

[B6] Droeser RA, Hirt C, Viehl CT (2013). Clinical impact of programmed cell death ligand 1 expression in colorectal cancer. Eur J Cancer.

[B7] Enkhbat T, Nishi M, Takasu C (2018). Programmed cell death ligand 1 expression is an independent prognostic factor in colorectal cancer. Anticancer Res.

[B8] Gado A, Ebeid B, Abdelmohsen A, Axon A (2013). Colorectal cancer in Egypt is commoner in young people: Is this cause for alarm. Alex J Med.

[B9] Gatalica Z, Snyder C, Maney T (2014). Programmed cell death 1 (PD-1) and its ligand (PD-L1) in common cancers and their correlation with molecular cancer type. Cancer Epidemiol. Biomarkers Prev.

[B10] El Jabbour T, Ross JS, Sheehan ChE (2017). PD-L1 protein expression in tumour cells and immune cells in mismatch repair protein-deficient and -proficient colorectal cancer: the foundation study using the SP142 antibody and whole section immunohistochemistry. J Clin Pathol.

[B11] Guo L, Lin Y, Kwok HF (2017). The function and regulation of PD-L1 in immunotherapy. ADMET DMPK.

[B13] Hirsch FR, McElhinny A, Stanforth D (2017). PD-L1 immunohistochemistry assays for lung cancer: Results from phase 1 of the Blueprint PD-L1 IHC assay comparison project. J Thorac Oncol.

[B14] Inaguma Sh, Lasota J, Wang Z (2017). Clinicopathologic profile, immunophenotype, and genotype of CD274 (PD-L1)-positive colorectal carcinomas. Mod Pathol.

[B15] Ioannou M, Paraskeva E, Baxevanidou K (2015). HIF-1α in colorectal carcinoma: Review of the literature. J Buon.

[B18] Kim JH, Park HE, Cho N-Y, Lee H, Kang GH 2016): Characterization of PD-L1-positive subsets of microsatellite-unstable colorectal cancers. Br J Cancer.

[B19] Kwon HC, Kim SH, Oh SY (2010). Clinicopathological significance of p53, hypoxia-inducible factor 1 alpha, and vascular endothelial growth factor expression in colorectal cancer. Anticancer Res.

[B20] Lee LH, Cavalcanti MS, Segal NH, e al (2016). Patterns and prognostic relevance of PD-1 and PD-L1 expression in colorectal carcinoma. Mod Pathol.

[B21] Liu R, Peng K, Yu Y (2018). Prognostic value of immunoscore and PD-L1 expression in metastatic colorectal cancer patients with different RAS status after palliative operation. Bio Med Res Int.

[B22] Masugi Y, Nishihara R, Yang J (2016). Tumor CD274 (PD-L1) expression and T cells in colorectal cancer. Gut.

[B23] Muenst S, Schaerli AR, Gao F (2014). Expression of programmed death ligand 1 (PD-L1) is associated with poor prognosis in human breast cancer. Breast Cancer Res Treat.

[B24] Patel SP, Kurzrock R (2015). PD-L1 expression as a predictive biomarker in cancer immunotherapy. Mol Cancer Ther.

[B25] Phillips TH, Simmons P, Inzunza HD (2015). Development of an automated PD-L1 immunohistochemistry (IHC) assay for non–small cell lung cancer. Appl Immunohistochem Mol Morphol.

[B26] Rebelatto MC, Midha A, Mistry A (2016). Development of a programmed cell death ligand-1 immunohistochemical assay validated for analysis of non-small cell lung cancer and head and neck squamous cell carcinoma. Diagn Pathol.

[B28] Rosenbaum MW, Bledsoe JR, Morales-Oyarvide V, Huynh TG, Mino-Kenudson M (2016). PD-L1 expression in colorectal cancer is associated with microsatellite instability, BRAF mutation, medullary morphology and cytotoxic tumor-infiltrating lymphocytes. Mod Pathol.

[B29] Scognamiglio G, De Chiara A, Di Bonito M (2016). Variability in immunohistochemical detection of programmed death ligand 1 (PD-L1) in cancer tissue types. Int J Mol Sci.

[B30] Topalian SL, Hodi FS, Brahmer JR (2012). Safety, activity, and immune correlates of anti-PD-1 antibody in cancer. N Engl J Med.

[B31] Valentini AM, Di Pinto F, Cariola F (2018). PD-L1 expression in colorectal cancer defines three subsets of tumor immune microenvironments. Oncotarget.

[B32] Wang L, Ren F, Wang Q (2016). Significance of programmed death ligand 1 (PD-L1) immunohistochemical expression in colorectal cancer. Mol Diagn Ther.

[B33] Xing X, Guo J, Wen X (2017). Analysis of PD1, PDL1, PDL2 expression and T cells infiltration in 1014 gastric cancer patients. Oncoimmunology.

[B34] Zhong G, Peng Ch, Chen Y (2018). Expression of STING and PD-L1 in colorectal cancer and their correlation with clinical prognosis. Int J Clin Exp Pathol.

